# A Canadian qualitative study exploring the diversity of the experience of family caregivers of older adults with multiple chronic conditions using a social location perspective

**DOI:** 10.1186/s12939-016-0328-6

**Published:** 2016-03-02

**Authors:** Allison Williams, Bharati Sethi, Wendy Duggleby, Jenny Ploeg, Maureen Markle-Reid, Shelley Peacock, Sunita Ghosh

**Affiliations:** McMaster University, School of Geography and Earth Sciences, 1280 Main Street West, Hamilton, ON L8S 4 K1 Canada; University of Alberta, Faculty of Nursing, 11405 87 Avenue NW, Edmonton, AB T6G 1C9 Canada; University of Saskatchewan, College of Nursing, 104 Clinic Place, Saskatoon, SK S7N 2Z4 Canada

**Keywords:** Elderly, Multiple chronic conditions, Informal caregivers

## Abstract

**Background:**

A little-studied issue in the provision of care at home by informal caregivers is the increase in older adult patients with chronic illness, and more specifically, multiple chronic conditions (MCC). We know little about the caregiving experience for this population, particularly as it is affected by social location, which refers to either a group’s or individual’s place/location in society at a given time, based on their intersecting demographics (age, gender, education, race, immigration status, geography, etc.). We have yet to fully comprehend the combined influence of these intersecting axes on caregivers’ health and wellbeing, and attempt to do this by using an intersectionality approach in answering the following research question: *How does social location influence the experience of family caregivers of older adults with MCC?*

**Methods:**

The data presented herein is a thematic analysis of a qualitative sub-set of a large two-province study conducted using a repeated-measures embedded mixed method design. A survey sub-set of 20 survey participants per province (n = 40 total) were invited to participate in a semi-structured interview. In the first stage of data analysis, Charmaz’s (2006) Constructivist Grounded Theory Method (CGTM) was used to develop initial codes, focused codes, categories and descriptive themes. In the second and the third stages of analysis, intersectionality was used to develop final analytical themes.

**Results:**

The following four themes describe the overall study findings: (1) Caregiving Trajectory, where three caregiving phases were identified; (2) Work, Family, and Caregiving, where the impact of caregiving was discussed on other areas of caregivers’ lives; (3) Personal and Structural Determinants of Caregiving, where caregiving sustainability and coping were deliberated, and; (4) Finding Meaning/Self in Caregiving, where meaning-making was highlighted.

**Conclusions:**

The intersectionality approach presented a number of axes of diversity as comparatively more important than others; these included gender, age, education, employment status, ethnicity, and degree of social connectedness. This can inform caregiver policy and programs to sustain health and well-being.

## Background

With an aging Canadian population, informal caregiving has become increasingly important. Available research evidence suggests, with regards to care, remaining in one’s home is usually preferred by older adults themselves [1, 2], which is in keeping with the government’s intention to transfer the responsibility of care to families at home. Canada’s public health care system has undergone a great degree of restructuring [3, 4] with an ever growing increase in care provided in the community, in the homes of families and friends of those requiring care [5]. This transfer of care responsibility has shifted what were formally ‘medical’ tasks from care professionals - such as nurses and doctors, to family/friend caregivers. The majority of the caregiving tasks, from personal care to medical procedures, provided to community-based elderly adults with MCC, are now the responsibility of family caregivers, with only complementary services (1–2 hours a week) provided by the health care system. Long-term institutional care is only available for those who can no longer manage at home, is costly and often requires the family caregiver to play a central role in service delivery. Consequently caregiver strain is a growing concern.

An important and little-studied issue in the provision of care at home by informal caregivers is the increase in older adult patients with chronic illness, and more specifically, multiple chronic conditions (MCC). Gilmour and Park [6] determined that of all community-living older adults in Canada, 33 % have MCC. Providing informal care for these older adults can be particularly challenging in view of their high use of healthcare services, high risk for adverse events and impaired ability to self-manage their own care [7–9]. Further, the intensity of healthcare use has a direct relationship with the number of chronic conditions [7, 10]. Finally, having MCC can impair patients’ ability to adhere to treatment and self-manage their care, which increases their dependency on family caregivers and the probability of adverse health outcomes [11].

Sustaining family caregivers and maintaining the health of family caregivers is critical, yet we know little about the caregiving experience for this population, particularly as it is affected by social location. Social location refers to either a group’s or individual’s place/location in society at a given time, based on their intersecting demographics, such as age, sex, economic class, sexual orientation, gender, education, race, immigration status, geography, etc. [12–15], and is often examined using an intersectionality framework. An intersectionality framework understands that social locations are intertwined, and unable to be separated. Such a framework is interested in equity and social justice and understands social location to be shaped by the influences of interacting and mutually constituting social processes and structures, impacted by power, time and place [16–18]. We have yet to fully comprehend the combined influence of social location on people’s health, and have little indication of how their health may change over time given the dynamic nature of caring for older adults with MCC. We attempt to do this in this paper, by answering the following research question: *How does social location influence the experience of family caregivers of older adults with multiple chronic conditions?* Throughout this paper “caregiver” refers to family/friend/neighbor informal caregiver (and not formal, paid caregivers).

### Literature review

The growing population living with MCC has a great impact on the family caregivers who provide care for them. Family caregivers provide up to 80 % of the care for community-living older adults with MCC, undertaking the majority of the costs and burdens associated with caregiving [19]. Although some family members are happy to care for a loved-one, caregiving unfortunately often results in sacrifices to their own health and well-being, with the level of caregiver strain shown to have a direct relationship with the number of chronic conditions the older adult has, increasing negative health outcomes and health service use in caregivers [20].

With respect to older adults with specific chronic diseases, those that had diseases which involved declined cognitive ability, such as dementia, required particularly intensive caregiving from family caregivers. For example, patients with dementia (i.e. Alzheimer’s disease), which is usually characterized by significant reduced memory loss, communication deficits, or even complete loss of functional daily activities, often require a constant caregiver [21]. The literature suggests that those who care for care recipients with cognitive impairment are at elevated risk of experiencing caregiver stress or burden [22]. Further, caregivers of patients with dementia reported high burden [23], increased depression and anxiety [24, 25], lowered level well-being, and poor physical health [23]. Among caregivers of older adults with various medical issues, those caring for recipients with dementia have been found to experience more negative mental and physical health outcomes, such as strain [23]. On the other hand, it is important to note that family caregivers in difficult situations (e.g., such as the context of dementia) can and do experience positive consequences (i.e., have the opportunity to give back or discover personal strength) related to caring for a loved one [26].

Existing research evidence has also shown that the health status of the care recipient plays an important role in caregiver mental health. In care recipients with Alzheimer’s Disease, patient cognitive status has been found to be a significant predictor of objective burden for caregivers (which includes items such as caregivers time to themselves, time to spend in recreational activities, and personal privacy) [27], as well as depression [28]. Similarly, overall functioning ability of care recipients has also been associated with increased burden for caregivers [28, 29]. In addition, research evidence suggests the status of the caregivers’ physical health, mental health [28, 30, 31], as well as demographic variables - such as older age, and being female, increased their risks of caregiver depression [28, 32], and anxiety [25]. One especially important connection with the mental health of caregivers is the available research on family dynamics [33]. High family cohesion has been associated with less caregiver burden and depression [33], and family conflict has been associated with increased caregiver depression and anger.

Sustaining caregivers and maintaining the health of caregivers is critical given that our health system relies on family caregivers as the backbone of care, yet we know little about the caregiving experience for this population, as it is affected by social location. Social location refers to either a group’s or individual’s place/location in society at a given time, based on their intersecting demographics (age, sex, economic class, sexual orientation, gender, education, race, immigration status, geography, etc. [12–15]. We have yet to fully comprehend the combined influence of these intersecting demographics on people’s health, and have little indication of how they may change over time given the dynamic nature of caring for older adults with MCC. Further, a social location or diversity lens would allow the identification of vulnerable caregivers, which can then better supported in these often complex and potentially stressful or burdensome situations; we attempt to do this in this paper.

With respect to informal caregivers of persons with multiple chronic conditions, there are some clusters of literature related to informal caregiving and a number of variables of diversity or social location, such as gender, age, education, geography, and social connectedness. However, most of this literature is focused on caregiving of persons with single not multiple conditions. The literature review is organized around these variables of diversity, beginning with gender.

#### Gender

Although there has been a rise in male caregivers, women still provide the majority of informal care to older adults [34]. As a result, the effects of care giving can differ between genders. In terms of well-being, women are more likely to report lower well-being or health status, regardless of the condition of the care recipient [32, 34, 35]. Specifically, poorer physical health is found among dementia caregivers [32] and negative psychological health is found among stroke caregivers [35]. Male caregivers, on the other hand, often have better well-being, both physically [36] and mentally [36, 37]. Women are more likely to experience symptoms of depression and caregiver burden, especially when caring for persons with dementia [32, 36, 38–41]. Women caregivers also tend to be at a greater risk for co-morbidities and chronic illnesses, regardless of the care recipient’s disease [42, 43].

With respect to seeking outside help for care giving tasks, men are more likely to pursue help from others [36, 37, 41] when caring for persons with dementia, while women are more likely to take all of the responsibilities of caregiving upon themselves [32, 41, 44]. However, women caregivers are more likely than men to have social support [34, 38, 45], while men are at greater risk for social isolation [42].

#### Age

Older caregivers are at greater risk for experiencing burden compared to their younger counterparts [46–50], when caring for persons with either stroke or dementia. Older caregivers are also more likely to be living with chronic illnesses themselves; however the prevalence of developing a chronic illness correlates more strongly with younger dementia caregivers when compared to the general population [43]. Thus there appears to be a significant correlation between age and burden.

#### Education

The level of caregiver education can impact caregiving. For example, dementia caregivers with a lower level of education are more likely to experience symptoms of depression than caregivers with a higher education level [29]. Higher educated caregivers of persons with dementia or stroke are more likely to experience a better quality of life and satisfaction [51], and better physical [52] and mental health [53]. Surprisingly, dementia caregivers who are more highly educated were found to experience a greater burden of caregiving [54]. Finally, stroke caregivers with lower education are more likely to experience feelings of fear and isolation [55], likely due to the fact that they lack information about and the initiative to seek social support services.

#### Geography

The location in which a caregiver lives in relation to the care recipient as well as care services can also influence their experiences of care giving. For example, Gort et al. [56] found a statistically significant relationship between dementia caregiver collapse and not living with the care recipient. This indicates that caregivers are more likely to experience collapse when they are living apart from their care recipient. In addition, caregivers who live a great distance from care services have a hard time accessing them [57]. Rural caregivers, especially, cannot conveniently visit the doctor and hospital and therefore are more likely to experience negative physical symptoms [58, 59]. Geographical distance has also been found to influence families to place loved ones with dementia in long-term care facilities or homes [60]. Families living farther away from the older adult with dementia are more likely to place them in a long-term care facility, rather than choose to provide informal care.

#### Social connectedness

The degree of social connectedness can have a great impact on the experiences of care giving. Frequent sources of social support for stroke and dementia caregivers include colleagues, neighbours, and friends [61, 62]. A strong negative correlation has been found between depression and social support, indicating that caregivers who lack a social support network are more likely to experience symptoms of depression [38, 63–66]. This association is mainly found among stroke and dementia caregivers.

In general, caregivers who have high levels of social support report higher levels of well-being and general health [65]. Caregivers who are also spouses tend to experience greater consequences socially. Hong and Kim [67] found that caregivers of their spouses with dementia rated themselves as having less social support and poorer physical health than caregivers who were not spouses of the care recipient. Tang and Chen [68] studied stroke caregivers and found a similar association, indicating that spouses who were satisfied with their social supports also had a better perceived health status. Caregivers who have a strong social support network are more likely to be satisfied with their role as a caregiver [69], have a positive attitude towards caring for dementia patients [70] or find meaning in care giving [71].

Little is known about how these variables representing social location interact. This qualitative study explored these parameters to enhance understanding of experiences of caregiving for older adults with MCC, at the intersection of these social locations. This is particularly needed given that none of the studies noted above considered caregiving for a person with MCC, but rather focused on only a single condition. As far as we know, this is the first qualitative study to explore the influence of social location on caregivers of older adults with MCC.

## Methods

**Fig. 1:**
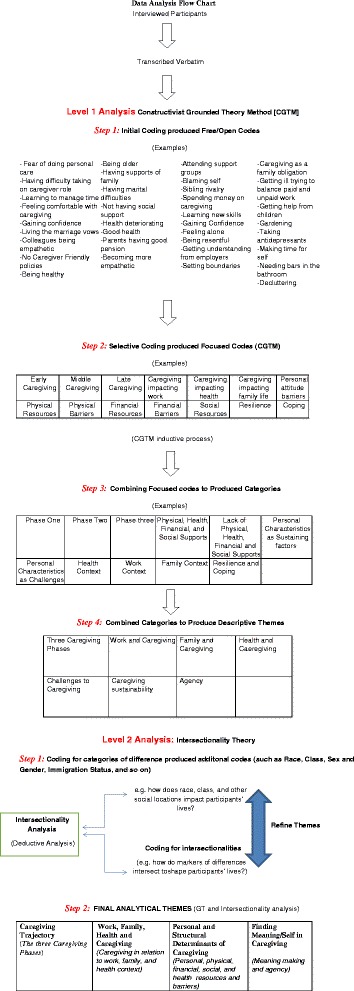
Data Analysis Flow Chart

The data presented herein is a thematic analysis of a qualitative sub-set of a large two-province study conducted using a repeated-measures embedded mixed method design [72]. Following Research Ethics Approval from both McMaster University and University of Alberta, caregivers were sampled from the two provincial jurisdictions of Alberta and Ontario. Inclusion criteria for the study were: a) informal caregiver, whether family or friend (aged 18 years or older) of an older adult (aged 65 years and older) with MCC living in the community; b) care recipient has three or more chronic conditions and was diagnosed with either dementia, diabetes or stroke – three common conditions for older adults - in the last 6 months prior to participating in the research; c) caregivers are English speaking. A multi-pronged snowball recruitment strategy was employed. Although not reported on here, all participants (*n* = 294) in the sample participated in two survey interviews six months apart; these survey interviews were held either face-to-face or over the telephone. A sub-set of 20 survey participants per province (*n* = 40 total) were invited to participate in a third qualitative stage following the second survey. These participants were purposively sampled to represent the various axis of diversity chosen for the intersectionality analysis. This qualitative stage provided an in-depth understanding of how the experience of caregiving was impacted by the multiple determinants of interest, while also probing the dynamic nature of caregiving for older adults with MCC. Interviews were semi-structured, audio-taped and transcribed verbatim. Interviews averaged 1 hour in length. Participants were assigned a code to ensure their anonymity. The codes are reported as either AP (Alberta participant) or OP (Ontario participant), followed by their participant ID number. The questions were aimed to understand the overall experiences of caregivers taking care of someone with MCC. Examples of these questions include*: Please tell me about your experience as a family caregiver, caring for someone with many chronic conditions? What is the biggest challenge for you?* All transcribed data were imported into NVivo. The analysis emerged in three stages (see Fig. 1). For a detailed analytical process see Sethi [73].

In the first stage Charmaz’s [74] Constructivist Grounded Theory Method (CGTM) was used to develop initial codes, focused codes, categories and descriptive themes. In the second and the third stages intersectionality analysis [75–77] was used to develop final analytical themes (Fig. 1). To develop the final analytical themes attention was paid to how participants’ social locations such as age, education, gender, geography, ethnicity, etc. [12–15] intersected simultaneously to shape their caregiving experiences. Such analysis “extends beyond gender-specific and social determinants frameworks” and focuses “on a variety of multi-level interacting social locations, forces, factors and power structures that shape and influence human life” [18]. The variation across the acuity of the MCC experienced by the older adults being cared for clearly has an impact on the caregiving experience. Given the dissimilarity of the diseases and disease patterns of the older adults being cared for, the analysis presented herein has not accounted for the variation in the caregiving experience, as impacted by the diagnosed diseases but has, rather, combined the experience of caring for older adults with MCC, as defined above.

## Results

As outlined in Table 1, 19 male and 21 female informal caregivers consented to participate in the study. The respondents ranged in age from 18 to 90 years and lived in an urban Census Metropolitan Area region (*n* = 38). A Census Metropolitan Area (CMA) is defined as an ‘area consisting of one or more neighbouring municipalities situated around a core. A census metropolitan area must have a total population of at least 100,000 of which 50,000 or more live in the core,’ [78] (https://www12.statcan.gc.ca/census-recensement/2011/ref/dict/geo009-eng.cfm). Most participants were married (*n* = 22). Participants reported the following ethnicities: Caucasian (*n* = 31), Black (*n* = 1), Chinese (*n* = 3), South-East Asian (*n* = 1), and Other (*n* = 4). Nineteen participants had a university degree. At the time of the study only 13 were working for pay (either self-employed or employed outside the home, full-time or part-time). One participant reported an annual household income of less than Canadian $10,000, 14 earned between $10,000-$39,999, 9 earned between $40,000-$69,999, and the income of 10 families exceeded $70,000. When asked if their finances met their needs, participants reported their needs met as totally inadequately (*n* = 2), not very well (*n* = 7), with some difficulty (*n* = 9), adequately (*n* = 13), very well (*n* = 4), and completely (*n* = 5). The median number of chronic conditions in the care recipients was 7, with the mean being 6.983 (SD 2.94) and the range being 3–13. The greatest number of care recipients had dementia (*N* = 22), followed by diabetes (*N* = 16) and stroke (*N* = 13).

**Table 1:** Description of caregiver characteristics (*n* = 40) for all participants

Characteristics	*n*
Caregiver Age	
Less than 45 years	4
46-50	3
51-55	7
56-60	6
61-65	3
66-70	4
71-75	4
76-80	4
81 years and older	5
Caregiver Gender	
Female	21
Male	19
Marital Status	
Single	9
Married	22
Divorced/separated	6
Other (common-law)	3
Ethnicity	
Caucasian	31
Black	1
Chinese	3
South East Asian	1
Other	4
Level of Education	
No high school	2
High school diploma (or GED)	9
College, GCEP	10
University Degree	19
Employed?	
Yes	13
No	27
Self-employed?	
Yes	3
No	10
What is your employment type?	
Full-time	6
Part-time	5
Unemployed	27
No answer	2
What is your relationship to the care recipient?	
Husband/wife/life partner	18
Son/daughter	18
Parent	1
Other	3
What is your estimated annual household income?	
Less than $39,999	15
Greater than $40,000	19
Prefer not to answer	6
Do your finances meet your needs?	
Totally inadequately	2
Not very well	7
With some difficulty	9
Adequately	13
Very well	4
Completely	5
Residency/geography	
Urban	38
Rural	2

Overall, the findings suggest that caring for individuals diagnosed with MCC (rather than single conditions), had a multiplicity of challenges. First, the caregivers were never sure about which condition was causing discomfort to the care recipients (AP549; AP593; AP622; OP069). Further, such caregiving was also very ‘demanding’ as it required constant vigilance of multiple ongoing conditions (AP520; AP631; AP521; OP069; OP112; OP101). In addition, caregivers were unsure about what condition to focus on during a health crisis (AP550; AP622): *“When you see a new symptom or change in behaviour, you don’t always know where that, what’s that all about? Is it part of one of those conditions, or is it something else going on”* (AP622). A spouse adds:

“*[Wife] does have some other conditions, you know and that, I find, is very demanding from the point of view you’re never sure what is the cause and effect, you know? I always say, if I could just take care of the Alzheimer’s we might be okay, but these other things that she has, are they affecting the Alzheimer’s? So that it’s, to me, the progression of the disease, again, it causes other effects, you know? And so you have to figure out, you know, is that from the dementia or does she actually have another different problem? And that’s what the constant battle is and that’s, you know, the whole problem solving bit comes in all the time. What do I have to do here, you know? Do I have to get her to the doctor or is it going to go away and will it never go away, have I got to accept this situation?”* (OP069)

To further complicate the situation, care recipients were taking multiple medications to address their many chronic conditions (OP018; OP027; OP112); caregivers were fearful that medication(s) for one condition may conflict with the other medications being taken for other conditions (AP564; OP018; OP112). Related to this, participants noted that they needed to consult with multiple doctors (AP622: AP638) about the care recipient’s conditions. This task was often difficult: *“…a lot of the medical specialties, they don’t connect well with each other”* (AP622).

Individuals found caregiving for persons with dementia in combination with other chronic conditions as more arduous than caregiving for persons with other conditions (AP520; AP524; AP605; AP622; AP535; AP569; OP069; OP074). A caregiver with both parents diagnosed with dementia notes: *“…they’re struggling through the dementia, they’re struggling with each other, they don’t understand, you know, where they are sometimes, what day of the week it is”* (AP535). The personality change is another complex aspect of dementia/Alzheimer’s that participants found challenging: *“It’s more of the personality and the, who is this? Like identity. Yeah, this is my mom but … Who is this woman? Who has she become? I don’t identify her meaning a lot to me because that person is gone, right? It doesn’t mean that my mom doesn’t mean anything to me, but my mom is gone, so that person is gone”* (AP569).

There was an underlining essence in participants’ responses that, regardless of whether caregiving was undertaken willingly/voluntarily, they were unprepared to provide care for individuals with MCC; one caregiver powerfully expresses this sentiment,*“…you don’t like the demands that are placed on you. You feel almost like an untamed horse being put in a bridle or something [laughing] like it’s not very comfortable and you’ve got to get used to it”* (AP622).

A husband caring for his wife with MCC notes: “*You have no life. Your own life gets put on hold and the demands of this person get more and more as time goes by and so you have less and less to be yourself and that’s the biggest thing I’m most working on is to retain me in all this*.” (OP069)

The following four themes describe the overall study findings: (1) Caregiving Trajectory, where three caregiving phases were identified; (2) Work, Family, and Caregiving, where the impact of caregiving was discussed on other areas of caregivers’ lives; (3) Personal and Structural Determinants of Caregiving, where caregiving sustainability and coping were deliberated, and; (4) Finding Meaning/Self in Caregiving, where meaning-making was highlighted.

### Theme one: Caregiving Trajectory

Participants’ responses suggest that in their caregiving journey there were three caregiving phases. These include the initial, middle and late phases. Each of these phases is explained below:

#### Phase One - Initial phase

The initial phase represented the period of time when the caregiver first assumes the caregiving role: *“…if I was to say what was the worst part, it would have been that first six months, was the worst”* (AP605). For others, this period lasted for up to two years: *“The first, 2012 to 2013 Christmas was very tough because all of a sudden we had got her in this routine from July to December”* (AP605). Another observes: *“I think maybe the first couple of years that…I’d say that my mom had Alzheimer’s it was hard”* (AP569). Regardless of the duration of time, there was consistency in most participant responses that this stage was very intense in terms of learning about the caregiving role and being prepared to provide care for family members with MCC. Learning often included the challenges related to understanding of various medical-related issues since care recipients had more than one medical condition:

*“Medical, yeah, well I’m just trying to think of…when she first came home she had a g-tube, for instance and I had to learn, like how to flush that out and, like, how to, like, change the dressing in it every week and stuff. She doesn’t have that anymore*” (OP024).

In this stage participants had difficulty managing time and taking care of their health since they were overwhelmed with the demands placed on them for caring for someone with MCC. Participants were more likely to do everything on their own (AP550; AP569; AP622). A daughter who was taking care of her mother noted: *“Yeah, I think maybe the first, hmm, first couple of years…I was there almost every day and I just felt I couldn’t say no”* (AP569). Those who did seek respite and other supports had to endure a waiting period:

*“…in the beginning we didn’t really have the services that we needed, like healthcare services that we needed to take care of her (grandma) at home and that was pretty stressful…”* (OP024).

Some participants struggled emotionally when dealing with their new caregiving role as the person had multiple chronic conditions:

*“…at the beginning, it was really hard, ‘cause I was yelling at her, blaming her. And you know, it’s just very time consuming, and it’s hard, it is really”* (OP077).

Often there was a feeling of guilt:

*“Yeah, yeah, like as I said the first part, the first few years, depression, you just feel guilt, you sort of feel you’re giving up your whole life for them, everything stops, you feel resentment, you feel, oh, haven’t I helped you enough in my life, financially and…mostly financially, right?”* (AP569).

Participants who were caregiving for individuals with dementia had great difficulty, especially in the first couple of years, dealing with the care recipients’ loss of memory. A caregiver illuminates:

*“I think maybe the first couple of years that…I’d say that my mom had Alzheimer’s - it was hard because you’re attached to this identity as a daughter and what NO … when she started not recognizing us and our names.”* (AP569).

#### Phase two - middle phase

As participants moved into the second phase, they had learned to adjust to the changes in their life due to caregiving for individuals with MCC:

*“Yeah, you sort of…it’s difficult to adjust to this change…. But you know, gradually over time you adjust and yeah, you know, learn to more or less deal with it …”* (AP521).

In this phase they accepted their caregiving role: *“I think I’ve accepted the caregiver role, it just came at a weird time in my life”* (AP569). Another participant adds:

*“… it’s like when you have a new baby you feel resentful that suddenly your life’s not your own, but you get used to it, right? It is what it is and you’ve got to grow up, right?”* (AP622).

This phase highlighted the formation of some routine in their life (AP605). Caregivers understood that they needed help with their caregiving duties: *“I think perhaps one of the things that’s changed from when I first started to now is that I realized that I cannot do it all…”* (AP550). As they sought out supports [such as Personal Support Workers (PSWs) for personal care], they learned to balance caregiving with managing their personal and/or professional lives: *“I think another hardest struggle was hard to balance, and it’s a little bit more balanced out now ….”* (AP569). With the passing of time, life became more organized: “*I had at first, when I first started two years ago, I didn’t understand how I had to do everything. Now that I’ve got everything organized…*” (AP605). Being organized, developing routine, and regaining some balance lost in the initial phase provided opportunities for self-care (AP605; AP622).

#### Phase three - late phase

The late phase is characterized by the caregiver showing confidence in their caregiving duties: *“I think I’ve sort of grown into the role and I feel quite competent actually, at this point, in terms of the role that I'm playing”* (AP525). In addition, participants appear to accept the care recipient’s diagnosis in this phase (AP615; AP521; AP569). A participant states:

*“I guess really understanding and accepting that, you know, the memory thing normally it doesn’t initially sound like it’s a medical problem. It sounds more like it’s…you know, the person is just sort of careless and whatever. Surely you can remember that kind of thing, you get the idea? How come you can’t remember that?”* (AP521).

At the same time, they learned to set caregiving boundaries: *“I had to kind of tell my dad in a nice way, and I didn’t know how to do it without creating problems and so he kind of backed off a little bit*” (AP569).

In addition to accepting health care supports, such as home support services, participants joined support groups or caregiver associations to educate themselves about the disease (AP525; AP550; OP011). Some participants even educated others (AP569) and became advocates for the care recipient (AP638). A daughter advocating for her mother stated:

*“…everybody needs an advocate for them and so I am happy that I can do that for her, and I think every elderly person, well everybody in the hospital, every sick person kind of needs that”* (AP638).

There was not only acceptance of the care recipient’s diagnosis, but a willingness to speak about it with others: *“I think that, like I said, I’m not ashamed to talk about it openly, whereas I used to…. It’s a stigma, right?”* (AP569).

This final phase also seemed to portray participants’ understanding that there were some positive outcomes from taking on the caregiving role. For example, in the ‘initial phase’ participants were scared of health-related caregiving duties, but later gained health-related knowledge as a result of the caregiving role (OP027; OP033; OP093; AP545; AP622; AP564). A participant expresses his views:

*“First, first, yes, and I have learned a lot. Like I never knew how, what it takes or how to look after an elderly person, like can’t do anything for themselves, because there are ways you turn, the way you clean, the way you take care of them, the way you feed them, because she’s PEG [percutaneous endoscopic gastronomy]-tube fed and at first, just seeing the tubes, I was scared”* (OP033).

Another participant adds: *“I feel good … I am of some value to my parents”* (OP018).

### Theme two: work, family, and caregiving

This theme addressed the impacts caregiving had on participants’ work, family and health. This theme was further divided into three sub-themes: (a) Caregiving and Work/Career that highlights how employment impacted caregiving; (b) Caregiving and Family highlights the loss of intimacy and family conflicts resulting from the demands of caregiving; and (c) Caregiving and Health highlights the physical and mental health impacts on caregivers and loss of social networks due to the constant need for care as the care recipients had MCC*.* These are briefly discussed below.

#### Caregiving and work/career

Managing paid employment and caregiving work was taxing for the eleven caregivers who also were employed (including self-employed). One participant confided:

*“So you cry every night and it affects you because with the full time job I had, I closed the door and cried and [laughing] a couple of employees saw me and it’s embarrassing”* (AP569).

In a similar vein, an adult son describes his challenges in balancing paid work and caregiving:

*“I moved in in 2010, at the end of 2010, took time off of work. Inevitably dealing with both my parents made it difficult for me to return to work. Emotionally I wasn’t strong enough to go back to work and I basically stayed here to care for him, to be a support, you know, emotional, mental, physical support, while we waited for the inevitable….”* (AP546).

Female caregivers employed in paid and unpaid work were *“…squished between two places”*; (AP622); often working multiple jobs (AP622) that required them to compartmentalize work and home life to manage both (AP569). Many were managing multiple roles, that is, taking of the house, family and spouse (OP078).

As the frequency of emergencies and hospital appointments increased, it was harder for some participants to integrate paid work with caregiving commitments (AP593; AP638). For those caregivers who were employed, a wide range of strategies were used in order to best meet the demands of the caregiving role. Four decided to leave paid work due to the time commitments of caregiving (AP545; AP525; AP546; OP036). As working far away from home created challenges for a wife taking care of her husband, indicating the impact of geography (where one lives and/or works) in caregiving, she moved closer to be available to him on short notice (AP622). She notes:

*“I could be closer in case something happened to him, ‘cause weird things, like I’d come home to find tea towels burnt and stuff, scorched stovetops and stuff, but I’d come home to find out that nobody had been here all day, you know?”*(AP622).

Intersection of geography and ethnicity is implicated in an immigrant caregiver’s decision to relocate to help his mother and be closer to his siblings. Growing up in Uganda he was taught to take care of his parents, especially mothers. Thus, he felt a strong sense of responsibility to provide care to his parents. He relocated and moved in with his mother as the primary caregiver (AP622).

One participant (AP631) took early retirement, while others either took time off work (AP546), reduced their workload (OP013), or dropped down to part-time hours (AP638). Finally, one participant mentioned that they had to put their educational goals on hold (AP581).

The data suggested that the work culture (whether the supervisor and/or colleagues were empathetic and if there were workplace accommodations available for caregivers) was critical in the participants’ ability to remain employed:

*“Well, I’ve been lucky at work that my boss is very understanding [emotional]. The boss above her is not understanding, at all, and that’s a whole other horrible story [laughing]”* (AP638).

Similarly, another participant describes his work culture as accommodating his caregiving role:

*“Great employer, very understanding. I can phone my boss and say, 'I gotta run, it’s ten o’clock, I’ve got a problem at the old folks’ home,’ and [snap] you know, you pass off the on-call cell phone pager, whatever, you go, right?”* (AP535).

For one caregiver, without the introduction of a new ‘duty to accommodate’ policy, where Human Resources assist workers to meet their caregiving or other needs, she would have been unable to keep her job (AP622).

#### Caregiving and family

There were clear differences in responses between spousal and adult children caregivers with regards to the impact of caregiving on family relationships. For spouses, the challenges were mainly related to not being able to do those things they had done in the past as a couple (AP520; OP063; OP069; OP075). A husband notes:

*“The challenge is finding things to do together, that she can participate in and she doesn’t care to watch much television, not that that’s something you do together, other than sitting side by side, but the things we’d enjoy, like walking and traveling and concerts and so on, are no longer possible and her eyesight is affected and because of the breakdown in communication between her eyes and the brain”* (OP063).

There was also loss of physical/sexual intimacy in marital relationships due to MCC (AP549; AP605). A wife caregiver explains: *“He’s like a child that I help, you know? There’s no more physical relationship, there’s none [laughing]”* (AP549).

Findings from adult children caregivers reveal several conflicts. There were often struggles between parents (as care recipients) and adult children as caregivers, due to past unresolved issues (AP564; AP535; OP077). A caregiver observes:

*“I think in my mind, always thinking about the past and all the crappy things that happened between us and all the crappy things that my mom did. Like the bad things she did, and the things she should have done. I don’t know why I am thinking of all these of things, but…”* (OP077).

Adult children caregivers also reveal sibling conflicts with respect to the caregiving task itself (AP525; AP569; AP545; AP631; OP013). For example, there were family troubles when caregivers perceived that their siblings were not contributing equally to caregiving (AP593; AP569; AP550):

*“…it’s more the sibling, I wouldn’t call it rivalry, but the sibling uneven contribution; but if someone’s selfish and tight with their money, there’s nothing you can do about it. You can’t force your sibling”* (AP569).

Even in families where siblings made a collective decision about caregiving, there was always difference of opinions about what was best for the care recipient (AP567). Sometimes these differences of opinion caused deteriorated family relationships (AP631; AP546) and created health issues:

“*I’ve kind of, you know, had it out with my sisters and said, blah, blah, blah. Like I kept it in for a long time and I think that caused me, like, I don’t want to say chest pain, but added stress and pressure that I didn’t need”* (AP569).

#### Caregiving and health

Findings suggest that participants’ health deteriorated as a result of the physical and/or emotional demands of caregiving for a person with MCC (AP535; AP545; AP546; AP550; AP581; AP593; AP622; AP631; AP638; OP013; OP061; OP063; OP093; OP042; OP033; OP036; OP078). The following quote demonstrates the caregiving and health relationship:

*“I’m on anti-depressants but you can’t even tell [laughing]. Yeah, when my dad got sick and I just felt like I couldn’t cope. Crying all the time. I hate crying all the time. It’s so embarrassing. But I don’t know, if that’s just a trigger…”* (AP638).

Participants who had to deal with their own physical and/or mental health issues found caregiving particularly arduous (AP521; AP522; AP569; AP 593; AP605; AP615; OP013; OP063; OP074; OP077; OP055; OP101). A participant explains:

*“I think probably on my emotional and psychological health it’s (caregiving) had a huge impact. I’ve had depression since I was probably 11, diagnosed when I was 14 and that’s caused me issues over the years, and I find it really, really difficult. I don’t have the freedom I used to have to step back when I’m not functioning very well…”* (AP593).

The stress of caregiving for multiple care recipients took a heavy toll on the participants’ bodies (AP550; AP593). The issue of deteriorating health was particularly visible in *older* individuals taking care of their spouse (AP521; AP524; AP549; AP605; AP615). A caregiver to her husband observes:

*“About nine months to a year ago, my health started to go downhill and Home Care [Services], they just stepped in. On one of the trips to the hospital with [name of care recipient], there was a lady there from Home Care [Services] and she just, for some reason she just came in - she was talking to [care recipient] and she looked at me and she said, ‘I think you’re the one that needs Home Care’* [*laughing*] ” (AP524).

The following quote further highlights how age impacts caregiving experiences: *“I guess as we’re both growing older and he’s deteriorating…I’m not in good shape, so it’s getting harder and harder”* (AP622). Moreover, the lack of leisure time or time for self-care was clearly indicated in participant’s’ responses (AP535; AP545; AP546; AP549; AP605; AP622; OP013; OP069; OP112; OP033). Due to lack of time for social interactions participants were not able to optimize their social connectedness. A participant observes:

*“You have no life. Your own life gets put on hold and the demands of this person get more and more as time goes by and so you have less and less to be yourself”* (OP069).

Women who were sandwiched between taking care of their spouse and/or children, as well as their care recipient parent(s) had even less time for self-care and infrequent participation in social activities (AP569; AP631). A participant observed that even when she and her husband took some time off, ‘caregiving’ was always on the back of her mind (AP631). Likewise, an adult child adds: *“Even if I went to the movies, I would have to have my blackberry on for phone calls in case I was needed”* (OP013).

#### Theme three: personal and structural determinants of caregiving

The gendered nature of caregiving was particularly apparent in adult children’s responses:

*“I felt that it was expected and not asked. It was just, you’re a girl and girls care give and boys don’t. So, I definitely saw, like, oh, you know, the gender divide and the expectations”* (AP569).

A participant argues: *“He’s the darling little boy. That’s a hard thing too, that he seems to be the favourite [crying] when I do everything”* (AP638). From a male caregiver perspective, women were better caregivers as:

*“… to do this job you need to be compassionate and understanding, which are things that men aren’t usually blessed with, [laughing] and, but I’ve had to learn that …”* (OP069).

Another caregiver agrees:

*“I mean, being the son and also falling into a role that, you know… from my perspective, easier to a woman, I find it stressful, you know”* (AP581).

Men did “yard work” (AP638), helped fixing things around the house (AP550), and/or looked after the inheritance (AP535); it was generally the women who provided personal care. Often, there was discomfort for male caregivers to provide personal care to their mother (OP018), especially when there were cultural norms against it:

*“…it was difficult for me when she could not manage to, what you call, to change her dresses or at night, especially in the evening time, when she wanted to put on her nightgown and go to sleep and she wouldn’t do it and that was…I used to feel, how should I put it? Very sad because, what you call, culturally it was a no-no for a son in our culture, to do that for a mother”* (AP567).

A male Chinese participant provides an alternative cultural facet, suggesting that parents live with the son and not the daughter:

*“…it’s a culture thing; boys take care of parents, and girls…it’s not their responsibility… so I have a lot of, already a lot of expectations on me, but my parents sometimes don’t remember that, like, I’m not a machine”* (OP018).

Even though this participant was considered the primary caregiver of his parents, his mother would not allow him to give her a massage as it was culturally a “gender-sensitive” issue. In his own words*: “I’m a boy, yeah, she feels uncomfortable…. I’d like to but I cannot, yeah”* (OP018).

The findings suggest that attitudes and beliefs about caregiving was an important component in caregiving sustainability. For example, spouses’ commitment to marital vows was a sustaining factor for most spouses interviewed (AP520; AP521; AP524; AP545; AP549; AP615; AP622; AP605; OP011; OP061; OP063; OP075; OP095; OP027; OP078). The following quote reflects such commitment:

*“Well, you know, the marriage vows we took was, you know, said in sickness and in health, until death us do part. So when problems come up, you deal with them, you know? And you find the best solution you can. That’s just the way it is”* (AP521).

Similarly, for adult children/grandchildren, having a positive attitude towards caregiving helped them through trying periods (OP018; OP033; OP055; OP101). Furthermore, participants who, during their caregiving career, were generally active and took good care of their health by eating well, exercising, and/or taking time for self-care, indicated that caregiving did not negatively impact their health (AP519; AP520; AP521; OP112; OP033).

Financial health emerged as an important component of caregiving sustainability. While financial difficulties added to the weight of caregiving (AP524; AP546; AP549; AP550; AP569; AP581; AP593; AP615; OP013; AP622; OP018; OP074; OP077; OP027; OP033; OP036; OP078; OP101), having a financial cushion eased the burden of caregiving (AP519; AP521; AP525; AP567; AP605; AP631; OP069; OP093; OP112; OP055). Furthermore, decluttering and/or having an physically accessible housing situation (one level housing, bars in the washroom, etc.) made caregiving a safer and more comfortable experience for both caregivers and care recipients (AP519; AP521; AP615; AP631; OP042; OP112; OP033; OP055; OP078; OP101).

Possessing skills, such as being able to cook, or having some medical background (i.e., first aid instructor, social worker, etc.), and/or hobbies (e.g., gardening, wood working, etc.) (AP520; AP521; AP605; OP013; OP112; OP069; OP075) aided participants in coping with the caregiving role. Educating themselves about their care recipients’ MCC, either through the internet, reading, and/or attending programs (e.g. Alzheimer disease educational sessions), relieved some of the stress while gaining some control over the situation (AP519; AP520; AP535; AP622; OP069). Volunteering (AP535; AP545), attending support groups and/or day programs (AP519; AP524; AP525; AP581; AP605); and/or using humor (OP013) assisted participants in managing demanding times. Friends and/or family emerged as the most important sources of support and feeling socially connected helped caregivers meet the arduous demands of caring for those with MCC (AP520; AP524; AP535; AP538; AP550; AP521; AP546; AP567; AP569; AP593; AP605; AP615; AP622; AP631; AP638; OP013; OP093; OP024; OP036; OP101; OP055; OP078; OP101).

However, for immigrants without family and friends, caregiving were particularly lonesome for lack of interpersonal networks and weak social capital: *“…we’re immigrants, we don’t have brothers and sisters or cousins or nieces or nephews or anything. So it’s just myself to take care of him”* (AP524). Religion and/or spirituality provided comfort and meaning to life and assisted caregivers cope with caregiving stresses (AP521; AP545; AP546; AP561; AP605; AP567; AP631; OP113; OP061; OP063; OP077; OP095; OP027; OP112; OP033; OP101), as evident in the following quote:

*“Spiritually I have a lot of faith and I’ve accepted that I’ve been chosen to do this and with God, I put God in front, first and foremost. I think that is what keeps me doing it and keeps me going”* (OP033).

#### Theme four: finding meaning/self in caregiving

Even though there were many physical, mental, emotional, and/or financial challenges faced by caregiving participants, they were able to find meaning in their caregiving role. Spending time together brought caregivers closer to their care recipient (OP018; OP011; OP093; OP024). Family bonding is expressed in this long and persuasive quote:

*“…even though it’s very sad, it’s brought some good in the sense that my family members are less spoiled, like they know that they have to step up now, so it’s a good thing, and it’s kind of like, you know, it’s like a family bonding moment as well, because when you’re helping one person, when you all have the same goal, you learn by each other a little bit too. So like there are things like, very small things, like before we never used to eat dinner together and now we do, right?”* (OP101).

Participants indicated that caregiving made them more empathetic (OP011; OP112; AP549; AP567). An adult child expresses gratitude:

*“I mean they’re thankful for what I do, but I also feel like I also have them to thank for a lot of different things, so I kind of see it as like kind of giving back…”* (OP101).

Similarly, other adult children caregivers expressed a sense of gratitude in being able to give back to their parents (OP033; AP638; AP525; AP535), and that by spending quality time with their parents (AP546; AP567; AP525; AP550) caregiving enhanced family cohesiveness by creating a *“…sense of togetherness as a family”* (AP567). As noted by an adult daughter:

*“I think one of the things that happened as a result of caregiving, was that walking away and sort of taking distance was not as optional as it had been, and so we just needed to stay through some difficult times together and there was probably a period of about close to ten years where that was actually a huge benefit in our relationship”* (AP593).

For male spouses in particular, it seemed that for a long time they had been at the receiving end of the benefits of marriage and now, for the first time in their life, were engaged deeply in their offering of benefits:

*“I think it’s enhanced my life overall, actually, by being so close to the center, [laughing] rather than out on the periphery somewhere, receiving all these benefits [laughing]*” (AP519).

A male spouse experienced pride in caregiving:

*“I think that as a caregiver, your role, as benefitting somebody that can’t do it themselves, gives you a little bit of pride to say, hey, I can do this. I’m capable of doing this, and I shouldn’t reject it”* (OP027).

Another spouse offered insights about ‘new life-enhancing learning’ that had resulted from caregiving: *“…a lot about housekeeping [laughing] like how to do laundry and learning a bit about how to cook and prepare meals”* (AP519). Out of necessity, women too learned to do things that their husbands always took care of:

*“I know I’ve learned things like how to take taps off and fix them and all those kind of things; [laughing] certainly household skills. There’s nothing I can’t do around the house anymore. I know I take care of everything now and I’m very proud of it”* (AP524).

A male participant is convinced that caring for his wife and staying active improved his health:

*“I’ve never been in better health [laughing] as far as energy and you know, alertness and stuff .... Because now I have to structure that part of it before the caregiving commences, actually”* (AP519).

## Discussion

Recognizing the diversity of caregivers who participated in the study, a number of commonalities were shared. First, caregivers shared the many common challenges of caring for a relative with MCC ranging from pharmaceutical interactions through to the lack of intra-professional communication amongst health care specialists. Further, all participant caregivers’ experiences contributed to the understanding of the three phases of the caregiver trajectory. Themes 2, 3 and 4 all highlight the many differences experienced by the family caregivers interviewed. These differences bring to light the importance of social location, which refers to either a group’s or individual’s place/location in society at a given time, based on their intersecting demographics (sex, gender, age, education, income, employment status, culture, geography, social connectedness, and other determinants, such as geography, etc.) [12–15]. Certainly, as determined by the findings from the 40 interviews of caregivers caring for patients with MCC, a number of these axes of diversity were found to be comparatively more important than others; these include gender, age, education, employment status, ethnicity, and degree of social connectedness. For example, the findings clearly highlighted the gendered aspects of caregiving. Men were primarily responsible for technical aspects of care, while women performed personal care. The issue of deteriorating health was particularly visible in older caregivers, implicating age as a unique challenge for these individuals. There was some evidence that immigrant status played an important role in social connectedness. Due to lack of time for leisure and self-care, waning social connectedness was visible in most of the caregivers, however, immigrants were more vulnerable to loss of social network than those born in Canada. Ethnicity and geography were two other axis of diversity implicated in caregiver’s experiences. Employment status intersected with gender to marginalize women more than men. Marital status was another axis of diversity in fulfilling caregiving roles (for example, findings showed that marital vows acted as an important value to willingly provide care to one’s spouse). Religious and/or Spirituality emerged as an important element of caregiver well-being. Furthermore, gender and ethnicity intersect powerfully in the aforementioned quote:

*“It’s a culture thing; boys take care of parents, and girls…it’s not their responsibility… so I have a lot of, already a lot of expectations on me, but my parents sometimes don’t remember that, like, I’m not a machine”* (OP018).

This division in labour confirms the traditional gendered roles in society, where men manage and women, due to their ‘nurturing’ traits, carry out the hands on care. Newman et al. [79] convincingly argue that “gender segregation of occupations, which typically assigns caring/nurturing jobs to women and technical/managerial jobs to men, has been recognized as a major source of inequality worldwide with implications for the development of robust health workforces”. While the central role women play in family caregiving is well known, men’s entry into caregiving work challenges the primacy of gender. This calls us to further explore gender in the caregiving experience, in order that we may be better able to understand the caregiving impacts on paid employment, finances and health, while fostering gender equality in caregiving work. When contemplating those who are most vulnerable to caregiver strain and burnout, it is equally important to gather an understanding of how gender intersects with other determinants of health (such as ethnicity and/or socioeconomic status) to shape caregivers’ experiences. For instance, in the current study, a ‘male’ Chinese participant is expected to take care of his parents; yet, even though he wants to he is not able to give his mother a massage.

We agree with Chappell et al. [80], with respect to the importance of examining the relationship that the caregiver has with the care recipient, as our results show that most spouses considered providing care to their partner as an integral part of the marital vows they had taken and so provided care willingly. This willingness to provide care to an ill spouse as a means to fulfill marital vows is similar to the findings by Hammond-Collins, Peacock and Forbes [81] wherein participants spoke about finding meaning and strength through their caregiving roles, particularly among husbands. However, child caregivers experienced stress and conflict with parents and/or their other siblings. Some women in our study occupied more than one caregiver role, providing informal care to the spouse, children, and parents.

## Conclusion

Chappell et al. [80] persuasively argue for using “the intersectionality framework for understanding co-occupancy of more than one status”. Much of the caregiver research has offered valuable insight into the impact of gender on caregiving. Intersectionality analysis has the ability to illuminate the unique experiences resulting from the intersection of diverse variables of diversity. It is clear from the current study that multiple and overlapping axis of diversity (such as age, ethnicity, employment status, geography, social connectedness, and immigrant status) influence the caregiving experience. Intersectionality can help to move beyond the focus on one disease at a time to gaining an understanding of intersecting and overlapping factors and concerns that individuals with MCC may experience. Such insight into the context-specific factors (meaning not taking it for granted that gender is the primary factor causing concern and examining the impact of other diversity axes) that are creating challenges is needed to help people their health and manage their chronic conditions. Certainly such results have implications for service provision, in that the needs of the most vulnerable can be prioritized.

There are several factors influencing this study. The first is that the sample was taken from a quantitative study, which meant that additional participants were not sampled to further inform the findings. As well they were mostly white with only a few that represent different ethnic groups. Further, all lived in urban areas. In order to challenge inequities and promote social justice it is integral that we understand within group differences (for example, how does the difference of a black caregiver who has a doctorate and earns $90,000/year differ from another caregiver who has high school and earns $20.000/year) and between group differences (how do caregiving experiences of South Asian caregivers differ from Black caregivers? Or how are lived caregiving realities experienced and shaped by geography, that is living in a well serviced urban region versus living in a medically underserviced rural area? Intersectionality theory is effective in investigating the impact of such lived realities based on multiple intersecting social locations (such as race, class, etc.) within a particular ethnic group and/or between different ethnic groups or between Caucasian and non-Caucasian participants [15]. Although such an analysis would have deepened the understanding of caregiving of older adults with MCC [15], it was not possible due to the lack of ethnic and geographic diversity across participants. Future research using an intersectionality framework must include participants representing different ethnic and cultural groups and those who live in rural areas. Such a multi-level analysis of intersecting factors (based on specific social locations such as ethnicity and/or geographic setting) is important to gather a comprehensive understanding of caregiver’s experiences and reveal the nuances of their complex lives. Specifically, intersectionality analysis in caregiving research offers the possibility of gaining critical insights of how caregiving issue are framed and informed by diversity. Further, as religion and/or spirituality were utilized as a coping mechanism, it would be worthwhile to explore the role of faith communities in supporting individuals and families dealing with MCC.

## Abbreviations

MCC: Multiple Chronic Conditions
CGTM: Constructivist Grounded Theory Method
PSWs: Personal Support Workers
